# Height Loss Is an Independent Predictor of Fracture Incidence in Postmenopausal Women: The Results from the Gliwice Osteoporosis Study (GO Study)

**DOI:** 10.3390/biomedicines11082231

**Published:** 2023-08-09

**Authors:** Wojciech Pluskiewicz, Piotr Adamczyk, Aleksandra Werner, Małgorzata Bach, Bogna Drozdzowska

**Affiliations:** 1 Department and Clinic of Internal Diseases, Diabetology and Nephrology, Metabolic Bone Diseases Unit, Faculty of Medical Sciences in Zabrze, Medical University of Silesia, 40-055 Katowice, Poland; 2 Department of Pediatrics, Faculty of Medical Sciences in Katowice, Medical University of Silesia, 40-055 Katowice, Poland; padamczyk@sum.edu.pl; 3 Department of Applied Informatics, Silesian University of Technology, 44-100 Gliwice, Poland; aleksandra.werner@polsl.pl (A.W.); malgorzata.bach@polsl.pl (M.B.); 4 Department of Pathomorphology, Faculty of Medical Sciences in Zabrze, Medical University of Silesia, 40-055 Katowice, Poland; bdrozdzowska@sum.edu.pl

**Keywords:** fracture prediction, height loss, postmenopausal women

## Abstract

Background. The aim of a longitudinal, retrospective study was to establish variables predicting fracture incidence over a decade. Methods. The study sample comprises a group of 457 postmenopausal women aged over 55 years, recruited from the database of an outpatient osteoporotic clinic. Several variables with potential influence on bone status, including the measurement of body height and hip bone densitometry, were collected. BMD at the femoral neck (FN BMD) was established using a Prodigy device (Lunar, GE, USA). Current body height was compared with the maximal historical body height in early adulthood, as reported by the patient. Results. Three hundred and ninety-four women did not have fractures during the follow up, and 63 subjects presented fractures. Subjects with fracture had lower FN BMD with a T-score of −1.86 ± 1.04 compared to −1.44 ± 0.89 in those without fractures (*p* < 0.001). Mean height loss (HL) was 3.47 ± 2.11 cm in fractured subjects and 2.50 ± 2.47 cm in unfractured ones, and differed significantly, *p* < 0.01. Fracture incidence was significantly related to age, rheumatoid arthritis, falls, and previous fractures. In the multivariable analysis using logistic regression, FN BMD, baseline fracture, and HL were identified as the significant predictors of fractures of follow up. Conclusions. Osteoporotic fractures in postmenopausal women were predicted by FN BMD, prior fracture(s), and HL easily established during physical examination.

## 1. Introduction

Osteoporosis is one of the most common chronic diseases in the elderly population.

The clinical significance of this disease depends on several factors. The number of patients is high and is likely to increase in the future. Osteoporosis has no obvious early symptoms and is therefore called the “silent thief of bones”, and due to the large number of people affected by it, it is referred to as the “silent epidemic”. The most common are involutional types of osteoporosis, e.g., postmenopausal (type 1) and senile (type 2). It should also be remembered that approximately 20% of all cases of osteoporosis have a secondary cause. Several chronic diseases of the endocrine system, liver, kidney, intestine or rheumatoid arthritis influence bone metabolism and status. In addition, prolonged use of many medications commonly used in daily practice may affect human skeletal status (glucocorticosteroids, aromatase inhibitors, anticonvulsants, loop diuretics, proton pumps blockers, heparin, or oral anticoagulants). The negative influence of different types of pharmacotherapy on bone metabolism is widely explored and discussed in the literature [1,2,3,4]. Fractures are the most important consequences of osteoporosis. Typical fractures in osteoporosis are caused by a fall from a standing height and are called “low trauma” fractures. However, some fractures, especially of the spine, can occur without obvious causes. The four fracture locations (spine, proximal femur, arm, and forearm) are usually classified as “major osteoporotic fractures”. Any fracture can cause serious damage to health, and fractures of the spine and proximal femur are the most damaging. In addition to the direct impact on human health, osteoporotic fractures play an essential role as a factor that increases the risk of subsequent fractures. For example, a fracture of the forearm doubles the risk of another fracture, and the most important fractures, e.g., of the spine or hip, increase the risk even by 4–6 times. Therefore, the primary goal of patient management is to avoid the first osteoporotic fracture. In addition to the purely medical consequences of the osteoporotic fracture epidemic, osteoporosis generates high costs for healthcare systems worldwide. The costs associated with surgeries for hip and/or vertebral fractures, as well as the prolonged rehabilitation required afterward, are of particular concern.

Some diagnostic procedures designed to reveal the process of bone loss are known and commonly used. Among them, bone densitometry is the most important. Scans of the lumbar spine and hip allow for the diagnosis of osteoporosis. In everyday practice, the most important thing is to determine the factors that increase the risk of fractures. As regards the risk of fractures, apart from osteoporosis assessed by densitometry, an important role is played by the so-called “clinical risk factors” and physical examination. The importance of clinical risk factors has been extensively studied and we know which ones increase the risk of fractures. Less obvious are the data from the physical examination.

Height loss (HL) and functional status leading to falls are among the most informative indirect signs of bone loss, and these data are relatively easy to collect in daily practice. Several papers have presented the potential role of HL [5,6,7,8,9,10,11,12,13,14,15,16,17]. In our recent studies, we evaluated the usefulness of HL in a large group of women from the GO Study [18,19]. We have observed that HL can be considered a useful marker of osteoporosis, with HL of 4–5 cm occurring in half of the patients with a typical osteoporotic fracture. It can therefore be assumed that HL may be a simple, easy to establish diagnosis, helpful in the everyday management of patients.

It can be assumed that the procedures recommended in everyday work with people suffering from osteoporosis should allow for precise determination of fracture risk. Diagnostic methods, including bone densitometry, skeletal radiography or laboratory tests, are not always available and add to the overall cost of healthcare systems. We need more diagnostic data to help practitioners. Of particular importance are diagnostic activities that can be easily performed during the examination of the patient. A simple variable such as HL is an example of such a finding with potential relevance to fracture risk assessment.

In the current longitudinal retrospective study, we attempted to determine which variables are significant predictors of ten-year fracture rates in a cohort of postmenopausal women in the GO study. The current study is a continuation of earlier reports on the role of HL as an indicator of fracture risk [18,19].

## 2. Materials and Methods

The initial study cohort comprised a group of 1881 women aged over 55, recruited from the database of an outpatient osteoporotic clinic in the South of Poland. The study was called the “Gliwice Osteoporosis Study” (GO Study). Women who were admitted clinic at least 10 years earlier were selected from the original database in order to establish the typical osteoporotic fracture incidence over a 10-year period. The number of such subjects was 567. For all of them, the necessary data with potential influence on bone status and metabolism, including clinical risk factors, measurement of body height, and bone densitometry, were collected using a structured questionnaire. To establish fracture incidence over the 10-year period, one investigator (W.P.) called all patients, and finally, data on fracture incidence for 457 patients were collected (80%).

BMD at the femoral neck (FN) was established using a Prodigy device (Lunar, GE, Chicago, USA). All bone scans were carried out by one experienced operator. The precision (CV%) of DXA measurements at FN was established at 1.6%, based on repeated measurements. Body height was always established on the day of DXA examination using a wall stadiometer (Seca, Hamburg, Germany). All body height measurements were performed by one technician. In order to calculate individual HL value, the current body height was compared with the historical maximal body height in early adulthood, as reported by the patient.

According to the previous decision of the Ethics Committee of the Medical University of Silesia (KNW/0022/KB/237/09, dated 1 December 2009), due to the study design, which is a retrospective analysis of pseudonymized data collected in medical records, the study protocol did not require separate consent from the review board.

### Statistics

Statistical analysis was performed using the Statistica 13.3 software (StatSoft, Tulsa, OK, USA). Mean values and standard deviations were used for descriptive statistics of continuous variables. The Shapiro–Wilk test was used to verify the normality of data distribution. Absolute values and percentages were given for qualitative variables. The Student’s *t*-test for independent samples or the Mann–Whitney U test (for data with and without a normal distribution, respectively) was applied for comparative analyses. Comparisons of qualitative features’ frequency were performed using the chi-square test. Logistic regression was employed to evaluate the independent influence of the analysed factors on fracture incidence. To confirm the utility of considering HL as a diagnostic variable for the prediction of osteoporotic fractures, the ROC curve generated based on this attribute was compared, among others, to the ROC curve based on FN T-Score. Various methods were used to find the optimal cut-off for HL that provides the best separation of cases with and without osteoporotic fractures, including the Youden index, the distance from the top left corner, and the intersection of sensitivity and specificity. The significance of results in all the statistical analyses was assumed at *p* < 0.05.

## 3. Results

The clinical characteristics of the whole study group and subgroups are presented in Table 1.

As mentioned in the description of the study group in the “Material” section, the inclusion criteria were: female gender, age over 55 at the start of the observation, and at least ten years of follow up in the outpatient osteoporosis clinic. Exclusion criteria included: male gender, age less than 55 years, shorter follow up, and lack of complete clinical data. The endpoints of the study were the occurrence of a fracture or the completion of the 10-year follow-up period without a fracture.

At baseline, 129 subjects reported prior osteoporotic fracture, and 328 patients did not experience fractures before the starting point of observation. The mean HL established at baseline was 2.63 ± 2.44 cm, and HL did not differ between women with previous fractures (2.87 ± 2.26 cm) and those without previous fractures (2.63 ± 2.53 cm).

During the observation period, 72 osteoporotic fractures were reported in 63 subjects. Fractures of the following skeletal sites were noted: forearm 20, spine 17, hip 13, ankle 9, arm 6, feet 5, rib and pelvis 1. In seven subjects, multiple fractures (two or three) were noted.

Subjects with new fractures were significantly older than not fractured patients (see Table 1; *p* < 0.05) and had lower T-scores for FN BMD: −1.86 ± 1.04 versus −1.44 ± 0.89 in unfractured ones, respectively (*p* < 0.001). Body mass, actual and previous historical height, and BMI did not differ between subgroups.

Mean HL was 3.47 ± 2.11 cm in fractured subjects and 2.50 ± 2.47 cm in unfractured ones, and differed significantly, *p* < 0.01.

Among qualitative features of clinical characteristics, rheumatoid arthritis, falls, and previous fractures were identified as factors significantly related to fracture incidence during the follow-up period. In women with rheumatoid arthritis, 50% noted a new fracture compared to 13.3% in the others (chi-square 6.7, *p* < 0.01). Among women who reported falls, 26.8% had new fractures, whereas only 12.0% of women without falls experienced new fractures (chi-square 9.1, *p* < 0.01). Finally, the new fracture incidence was at 23.3% in the subgroup with fracture(s) reported at baseline and only 10% in the subgroup without baseline fractures (chi-square 13.6, *p* < 0.001). There was no significant relation between new fractures and such factors as steroid use, smoking, and secondary reasons for osteoporosis.

For the final identification of factors with significant influence on new fracture incidence, the multivariable analysis by means of logistic regression was performed. All factors identified in univariable analyses, namely: age, T-score for FN BMD, HL, prior fractures, rheumatoid arthritis, and falls, were included in the primary model. In the final model, only three remained with statistically significant influence on fracture incidence in the follow-up period: baseline fracture, HL, and T-score for FN BMD. The odds ratio values of those factors for new fractures are presented in Table 2. The efficacy of this final model in terms of AUC (Area Under the ROC Curve) is 0.69 (95% CI 0.62–0.76).

**Figure 1:**
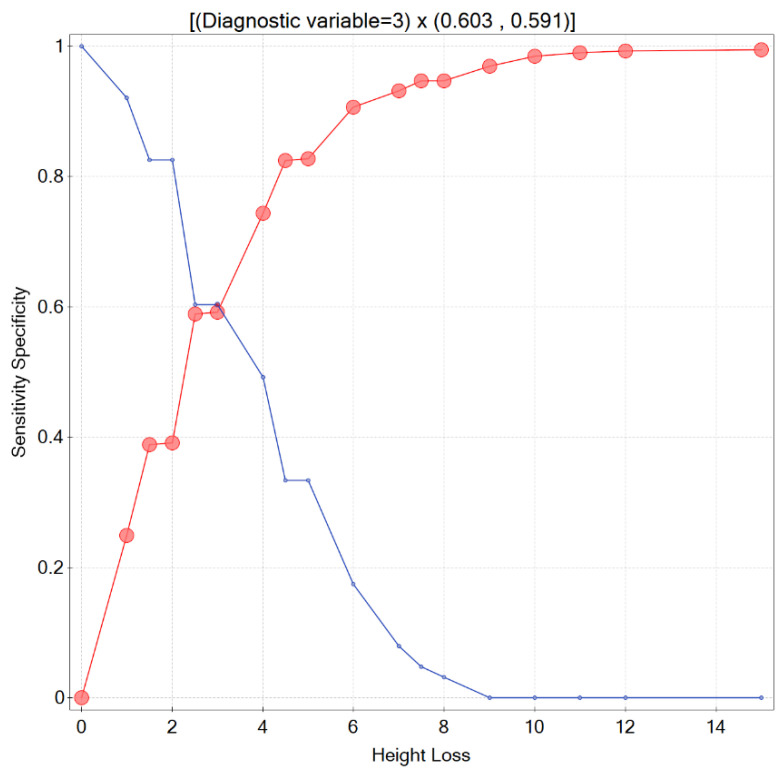
Sensitivity and specificity intersection graph.

Since in medical practice it is essential to know what degree of a patient’s height loss should raise a doctor’s concern and suggest ordering additional workup, this issue was thoroughly investigated in the presented study. The accuracy of the prediction expressed by the area under the ROC curve, obtained solely based on HL, was AUC = 0.648 (95% CI 0.58–0.72). The cut-off value determined by Youden’s criterion and the distance from the top left corner was for HL = 4 cm (sensitivity = 0.492 and specificity = 0.744). However, considering the sensitivity and specificity intersection graph, the localization of the point in which the value of both measures was simultaneously the greatest (sensitivity and specificity equalled 0.603 and 0.591, respectively) was for HL = 3 cm (Figure 1).

However, determining the optimal cut-off point for a particular variable, such as HL, is a complex task that should be carried out by medical professionals. It involves considering various factors, including the costs associated with different types of errors, such as false negatives and false positives, as well as the type of the disease [20]. The values of the sensitivity and specificity for the chosen HL thresholds are presented in Table 3.

**Figure 2:**
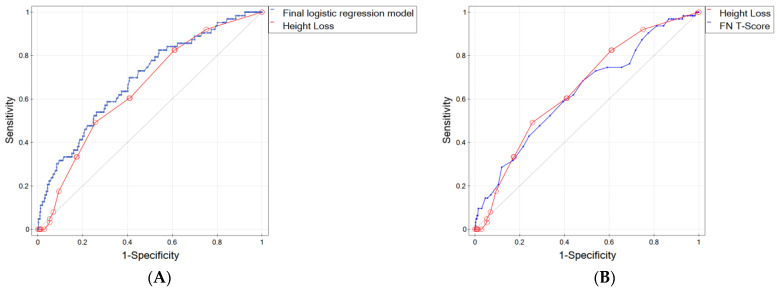
ROC curves for (**A**) final logistic regression model and height loss, (**B**) height loss and FN BMD T-Score. The diagonal line represents points where sensitivity = 1-specificity (i.e., it represents a diagnostic test that is no better than chance level).

As mentioned above, the accuracy of the prediction, expressed by the area under the ROC curve, was 0.69 (95% CI 0.62–0.76) for the proposed logistic regression model and 0.648 (95% CI 0.58–0.72) for the model based solely/only on HL (Figure 2A). To determine whether the AUCs of these two models were statistically significantly different, DeLong’s nonparametric test was performed. Based on the obtained *p*-value of 0.250766, we concluded that it is not possible to definitively determine which model better predicts osteoporotic fractures.

We also compared the ROC curve for HL as a potential fracture factor with the curve based on the FN T-Score, which is treated as a gold standard for the diagnosis of osteoporosis (Figure 2B), and found that the AUC for HL was better than for the T-Score by 0.016 (0.648 for HL vs. 0.632 for T-Score). Such an observation is of great importance because HL is easily measurable in daily practice. It does not require any specialized medical tests, thereby avoiding additional costs.

## 4. Discussion

The most significant finding of the current longitudinal retrospective study was the identification of height loss (HL) as a simple and easily measurable variable that significantly predicts fracture incidence over a 10-year follow-up period. Another two factors which also played a role in fracture prediction were: baseline femoral neck densitometric measurement and prior osteoporotic fracture. Unexpectedly, age and functional status expressed by falls did not influence fracture incidence in multivariable analysis. This unexpected finding should be further investigated. In daily practice with osteoporotic patients, we need to gather data useful for fracture prediction. Obviously, the most valuable are data which are easy to collect and cost-effective. HL is an example of such a variable. Based on current results, one may state that simple height measurement and interview, which reveal prior osteoporotic fracture, plus bone densitometry, provide reliable information for fracture prediction. As we mentioned earlier, the role of HL in osteoporotic populations was widely studied [5,6,7,8,9,10,11,12,13,14,15,16,17,18,19]. In several studies, the prospective observation was performed [7,8,9,10,11,12,16,17]. In the current study, we also performed a longitudinal observation. Therefore, the most interesting are comparisons with results in these studies. In an Australian DUBBO study, hip BMD, HL, and falls predicted fractures of the arm, forearm, and wrist [7]. In a study by Moayyeri et al., an annual HL of 1 cm was comparable to having a past history of fracture [8]. Other authors revealed that HL > 5 cm was associated with a nearly 50% increase in risk of hip fracture [9]. Contrary to this, in a long-term 17-year follow up in women, HL did not predict hip fracture [10]. However, in the same study, recent HL increased the risk of hip fracture. Other studies have shown that height loss (HL) plays a similar role in relation to spine fractures [11] and fractures in general [12]. In the Canadian study CaMos, in a group of 2588 women with osteopenia, increased fracture risk was influenced by low femoral neck BMD, prior fracture, HL, and self-reported worse general health condition [16]. Interesting data are provided in a Taiwanese study [21]. The authors compared nine different tools for identifying fracture/osteoporosis risk. Among several clinical risk factors for osteoporosis and densitometric and quantitative ultrasound measurements, HL was not considered a risk factor. We suggest that HL should be taken into account in attempts to predict fracture. Previously presented data shown by many authors suggest that HL may be an additional, significant risk factor. Also, our previous studies confirm the potential role of HL in the osteoporotic population [18,19].

The current study has some limitations: the study concerns only women, we performed longitudinal retrospective study instead of true prospective observation, and the population was not randomly recruited. Spine radiograms were not always available so some vertebral fractures might have been omitted. For this reason, our study design and material cannot be used to determine whether the HL is a fully independent predictor of fractures or a surrogate of a previous vertebral fracture. However, this does not undermine the demonstration of the practical clinical role of HL as a single, relatively easy to establish parameter of the clinical assessment of the patient. Due to the recruitment design, the analysis was carried out in a group with a relatively wide age range, so age may be a confounding factor in the analyses, as both HL and the fracture incidence are age-related. One should also remember that HL was established using maximal historical height reported by patients and such information cannot be reliably confirmed. However, study size, long period of the follow up, and baseline collection of vast number of factors with potential influence on fracture risk support the study results.

The results of the current study may also be an impulse to investigate future fracture risk using partially new data. The new tools developed to establish fracture probability or risk have been introduced since the middle of the first decade of the XXI Century. FRAX developed by J. Kanis and colleagues in Center Collaborating with the World Health Organization in Sheffield became the most popular one [22]. This tool allows presenting fracture risk expressed as probability limited by life expectancy. FRAX shows separately the probability of a hip fracture and major fractures (hip, spine, arm, forearm) in percentages. Another method is an algorithm designed and developed in Australia [23,24]. Based on long-term prospective observation of a great epidemiological cohort of males and females in Dubbo, the authors presented a nomogram called Garvan that was able to establish the risk of osteoporotic fracture for the hip or any skeletal site. Interestingly, only some risk factors are included in both mentioned diagnostic tools. Only age, prior fracture (for Garvan the role also plays the number of prior fractures), and femoral neck BMD were used in both algorithms. FRAX also takes into account the history of hip fracture in parents, current smoking, steroid use, rheumatoid arthritis, secondary osteoporosis, and alcohol intake. For the Garvan algorithm, the fourth risk factor is the number of falls in the last 12 months. The differences between FRAX and Garvan may be partially caused by different designs. More recently another algorithm called POL-RISK was developed and presented [25,26]. This tool is based on a 10-year observation of an epidemiologic female sample aged over 55 years. Factors established as those which influence fracture risk were the same as in the Garvan method (except that the number of prior falls was not included). All three methods are easily available on the websites; therefore, fracture risk may be quickly calculated and used in daily practice. We consider that the results of the current study suggest that HL may be added to the panel of potential baseline risk factors and improve the accuracy of fracture risk assessment. Such studies should be performed in the future.

To conclude, osteoporotic fractures in postmenopausal women were predicted by femoral neck densitometry, prior fracture, and HL. HL may be easily measured during the physical examination and should always be included in daily practice, especially in the health status assessment of elderly people.

## Figures and Tables

**Table 1:** Clinical characteristics of the whole study group and subgroups.

Variable	Whole Group *n* = 457	Fractured Patients *n* = 63	Non-Fractured Patients *n* = 394
Age (years)	64.21 ± 5.94	65.92 ± 5.61	63.94 ± 5.95 *
Body mass (kg)	71.25 ± 13.3	70.20 ± 13.63	71.41 + 13.24
Actual height (cm)	158.75 ± 5.97	158.38 ± 5.56	158.8 ± 6.04
Maximal height (cm)	161.4 ± 5.82	161.85 ± 5.27	161.33 ± 5.91
Height loss (cm)	2.63 ± 2.44	3.47 ± 2.11	2.50 ± 2.47 **
BMI (kg/m^2^)	28.25 ± 5.31	27.91 ± 5.82	28.32 ± 5.22

* significantly younger than fractured patients, *p* < 0.05. ** significantly smaller than in fractured patients, *p* < 0.01.

**Table 2:** Clinical factors of baseline characteristics identified in logistic regression analysis as significantly related to fracture incidence in the follow-up period.

Factor	Odds Ratio (OR)	Confidence Interval (CI)	*p* Value in Univariable Analysis	*p* Value in Multivariable Analysis
Previous fracture	2.31	1.31–4.07	<0.001	<0.01
FN BMD T-score	0.62	0.43–0.88	<0.001	<0.01
Height loss (cm)	1.14	1.02–1.26	<0.01	<0.05

**Table 3:** Values of the sensitivity and specificity for different HL thresholds.

HL (cm)	Sensitivity	Specificity
2	0.825	0.391
2.5	0.603	0.589
3	0.603	0.591
4	0.492	0.744

## Data Availability

Data are available on request.
